# Clinical implementation of the 3D 4K exoscope (Orbeye™) in reconstructive head and neck surgery

**DOI:** 10.1007/s10006-025-01342-6

**Published:** 2025-02-03

**Authors:** Elena Hofmann, Christian Doll, Steffen Koerdt, Cynthia Kurth, Max Heiland, Kilian Kreutzer

**Affiliations:** 1 Department of Oral and Maxillofacial Surgery, Charité – Universitätsmedizin Berlin, corporate member of Freie Universität Berlin and Humboldt-Universität zu Berlin, Augustenburger Platz 1, 13353 Berlin, Germany; 2 BIH Biomedical Innovation Academy, BIH Charité Junior Clinician Scientist Program, Berlin Institute of Health at Charité – Universitätsmedizin Berlin, Charitéplatz 1, 10117 Berlin, Germany

**Keywords:** Exoscope, Intraoperative microscope, Microsurgery, Microvascular anastomosis, Reconstructive head and neck surgery

## Abstract

**Purpose:**

To assess the clinical utility of the 3D 4K exoscope for reconstructive head and neck surgery.

**Methods:**

This retrospective study analyzed surgical details and complications with the use of the 3D 4K exoscope for microvascular reconstruction at a high-volume Department of Oral and Maxillofacial Surgery, compared to the use of a 2D microscope. Patients with oral cancer undergoing microvascular reconstruction were categorized into two cohorts based on the intraoperative use of the 3D 4K exoscope (Orbeye™, Olympus, Tokyo, Japan) or a conventional microscope (ZEISS S8 – OPMI Vario, Carl Zeiss AG, Oberkochen, Germany; Leica M680, Leica Mikrosysteme Vertrieb GmbH, Wetzlar, Germany) during a six-month study period, respectively. Outcomes were also compared between two time periods of the exoscope use to assess the learning curve over time.

**Results:**

The exoscope was applied for microvascular anastomosis in 55 surgical cases (cohort 1), and the conventional microscope was employed in 56 cases (cohort 2). The rates of postoperative complications within 14 days following the use of the exoscope were 14.5% (*N* = 8), compared to 16.1% (*N* = 9) in cohort 2. Analysis over time demonstrated a learning curve with the exoscope, reflected in a decrease in postoperative complications within 14 days from 22.7 to 9.1%.

**Conclusion:**

The three-dimensional camera system provides excellent and reliable intraoperative visualization in reconstructive head and neck surgery. Transitioning to this new technology did not lead to an increase in intra- or postoperative complications, but the successful implementation requires some experience with the device.

## Introduction

Since the first intraoperative use of the microscope in 1921 by Carl-Olof Siggesson Nylen, the microscope has evolved into an indispensable instrument in the operating theatre [1]. Magnifying loupes and a headlight may serve as a suitable magnification technique for the individual surgeon depending on the surgical context, but the microscope offers the additional advantage of enabling multiple team members to visualize the same surgical detail.

The use of a microscope has become a standard tool in microvascular reconstruction for head and neck cancer. The development of binocular microscopes paved the way for the implementation of three-dimensional imaging. In 2017, the Orbeye™ (Olympus, Tokyo, Japan), a 3D 4K exoscope, was introduced to the medical sector. Since then, the three-dimensional camera system has been implemented in various surgical specialties, including neurosurgery, among others [2–7]. The exoscope is designed to combine features such as magnification, stereoscopic vision, and illumination within a confined operating field. Unlike traditional microscopes that require eyepieces, the exoscope projects the magnified and illuminated surgical field onto one or several monitors, which the entire surgical team, including surgeons, assistants, scrub nurses, or observers, can view using special 3D glasses.

The potential advantages of the exoscope include its multi-component design, which relies on both the camera system and monitors, enabling enhanced maneuverability of the individual components and versatile positioning within the operating room [8]. Additionally, omitting the need for eyepieces and a fixed working distance may reduce the unphysiological flexion of the surgeon’s spine common during the use of the conventional microscope, potentially improving ergonomics for surgeons.

Recent studies have discussed their first experiences with the use of exoscope in the field of neurosurgery [4, 6, 7, 9] and have suggested a short learning curve [9]. However, studies on the clinical use, feasibility, and outcomes of the Orbeye™ remain limited. While the device shows promise in facilitating reliable microvascular anastomoses, even in challenging and less accessible areas, further research is necessary to validate its advantages and confirm its role as an alternative to conventional microscopes in head and neck surgery. This study adds to the growing body of literature by exploring the application of the exoscope in microvascular reconstruction for primary oral cancer and comparing its use to the conventional microscope.

## Patients and methods

### Ethics statement

This study was approved by the Ethics Committee of the Faculty of Medicine, Charité Berlin (EA1/044/23) and was conducted in accordance with the Declaration of Helsinki. Informed consent was waived due to the retrospective nature of the study and the analysis of anonymized clinical data.

### Study design

This retrospective study compared the use of the 3D 4K exoscope with the conventional microscope and assessed the learning curve using the exoscope by comparing the clinical experience with the Orbeye™ over time. This study included patients who received primary surgical treatment for oral cancer comprising microvascular reconstruction with the use of an operating microscope or exoscope during microvascular anastomosis in the Department of Oral and Maxillofacial Surgery at Charité – Universitätsmedizin Berlin at two locations, Campus Benjamin Franklin and Campus Virchow Klinikum.

**Fig. 1:**
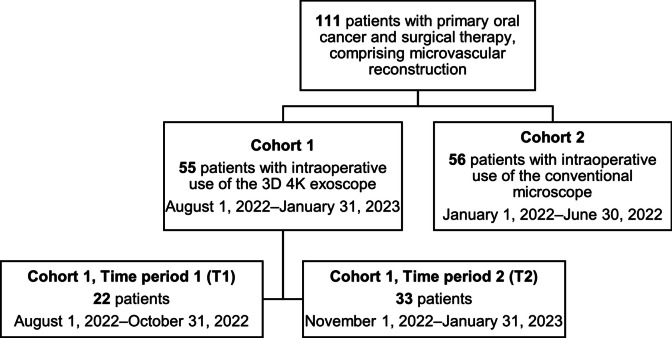
Flow diagram showing the patient cohorts eligible for analysis

Patients were categorized into cohorts 1 and 2 according to the exoscope or microscope use (Fig. 1). Cohort 1 included patients who underwent surgery between August 1, 2022 and January 31, 2023 using the 3D 4K exoscope (Orbeye™, Olympus, Tokyo, Japan). In order to assess the learning curve using the exoscope, outcomes were compared between two time periods. The first period (T1) was from August 1, 2022 to October 31, 2022, and the second time period (T2) between November 1, 2022 and January 31, 2023. Cohort 2 served as a control group and included patients who underwent surgery between January 1, 2022 and June 30, 2022 using a conventional microscope (ZEISS S8 – OPMI Vario, Carl Zeiss AG, Oberkochen, Germany; Leica M680, Leica Mikrosysteme Vertrieb GmbH, Wetzlar, Germany). The transition period from conventional 2D microscope to exoscope use (July 1–31, 2022) was excluded.

The cohort only included patients that underwent surgical procedures for primary squamous cell carcinoma located at the oral cavity. Patients with a previous diagnosis of a head and neck cancer were excluded. Patients that had previously undergone head and neck surgery or radiotherapy were also not eligible for inclusion. The process of patient inclusion is detailed in Fig. 1, modified after Consolidated Standards of Reporting Trials (CONSORT) [10]. Data were retrospectively analyzed regarding patient demographics, type of procedure, operative duration, as well as intra- and postoperative observations and complications according to the Clavien-Dindo classification [11]. Postoperative complications grade IIIb according to the Clavien-Dindo classification within 14 days following the primary surgical procedure and postoperative complications that occurred thereafter were retrospectively analyzed. All procedures were performed by senior surgeons of the Department of Oral and Maxillofacial Surgery familiar with microvascular surgery.

### Exoscope details

In contrast to the conventional microscope, the use of an exoscope eliminates the need for eyepieces and a fixed working distance. The Orbeye™ provides a 3D and 4K image, which can be visualized on one or more 55-inch or 31-inch monitors using specialized 3D glasses (Sony Corporation, Tokyo, Japan). The system allows an improved depth perception, an up to 26x magnification, and a field of view up to 171 mm. The device consists of a main unit and an extracorporeal telescope (exoscope) that contains a robotically assisted arm with a camera at its end. The exoscope can be easily steered manually in all directions. With the aid of a foot pedal, fine adjustments can be made. The near infrared image mode allows the visualization of vascular structures. Intraoperative 4K photo and video recordings can be made at any time in both 2D and 3D.

### Exoscope set-up in head and neck surgery

Unlike the conventional microscope, the exoscope setup comprises multiple components, including the camera system and wired monitors. Although the multi-component system requires substantial space in the back of the operating room, the exoscope itself, featuring a small camera mounted on a slim arm, occupies little space in the operating field. This compact design allows for quick transitions between macroscopic and microscopic procedural steps.

**Fig. 2:**
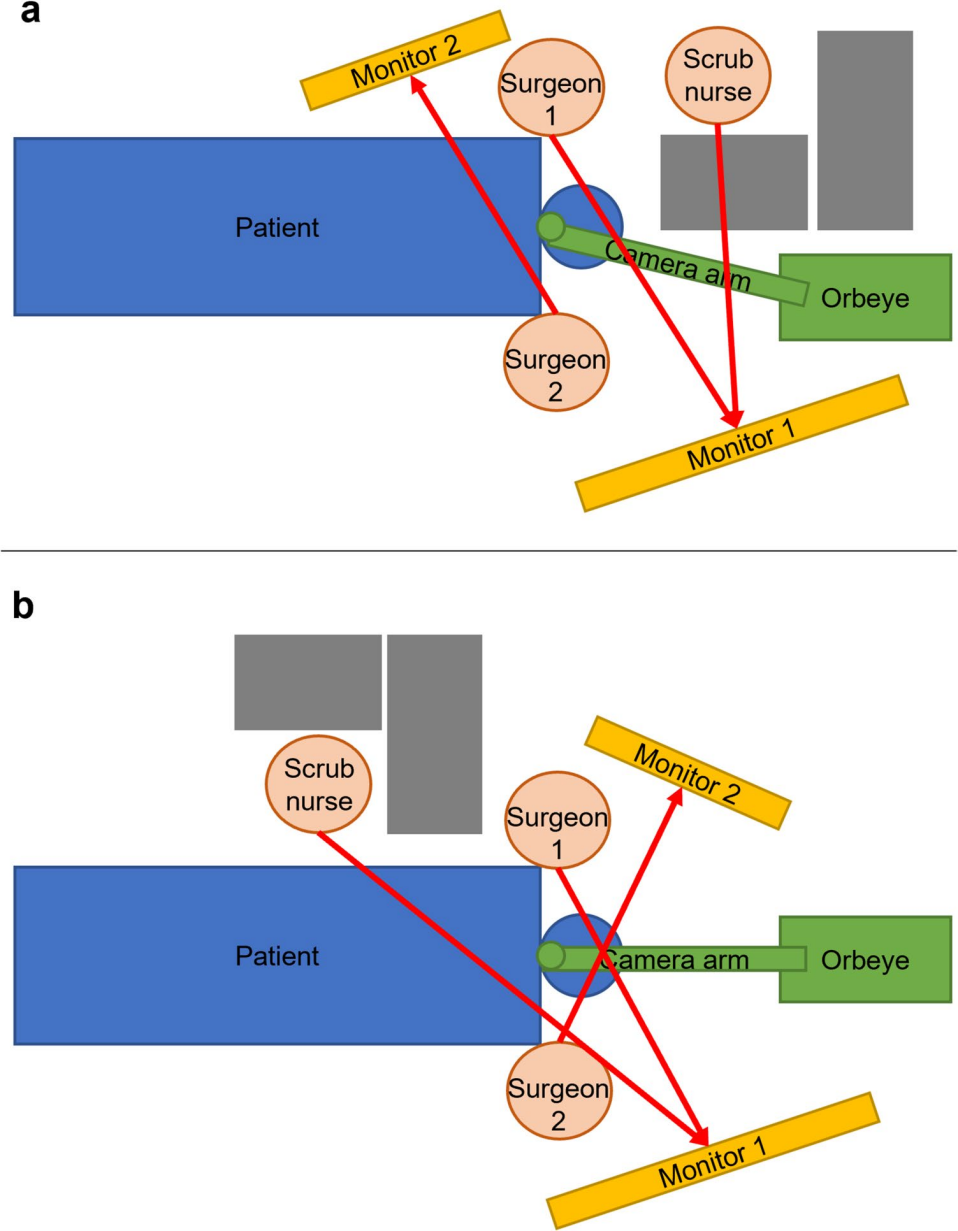
**a**,** b** Intraoperative set-up of the Orbeye™ 4K 3D system in head and neck surgery. Two options (**a**) and (**b**) for the exoscope set-up are illustrated. The first option (**a**) is the favored arrangement because it allows the shortest distance between the surgeons and the monitors, but the second option (**b**) may be applied depending on the standardized set-up. The camera system (green) is positioned cranially to the patient’s head. The surgeons sit on opposing sides left and right lateral to the head rest (blue). The monitors (yellow) are positioned straight ahead of each surgeon, so that the surgical field on the monitor can be visualized in an upright position. This set-up serves as an example for the performance of an anastomosis on the patient’s right side so that the main surgeon (surgeon 1) is seated on the right side of the patient and has a direct view of the main monitor (monitor 1) positioned on the opposite side of the patient. The red arrows indicate the surgeons’ and the scrub nurse’s viewing directions. The foot pedal, positioned underneath the head rest, facilitates easy adjustments of the focus, magnification, illumination, and camera direction

**Fig. 3:**
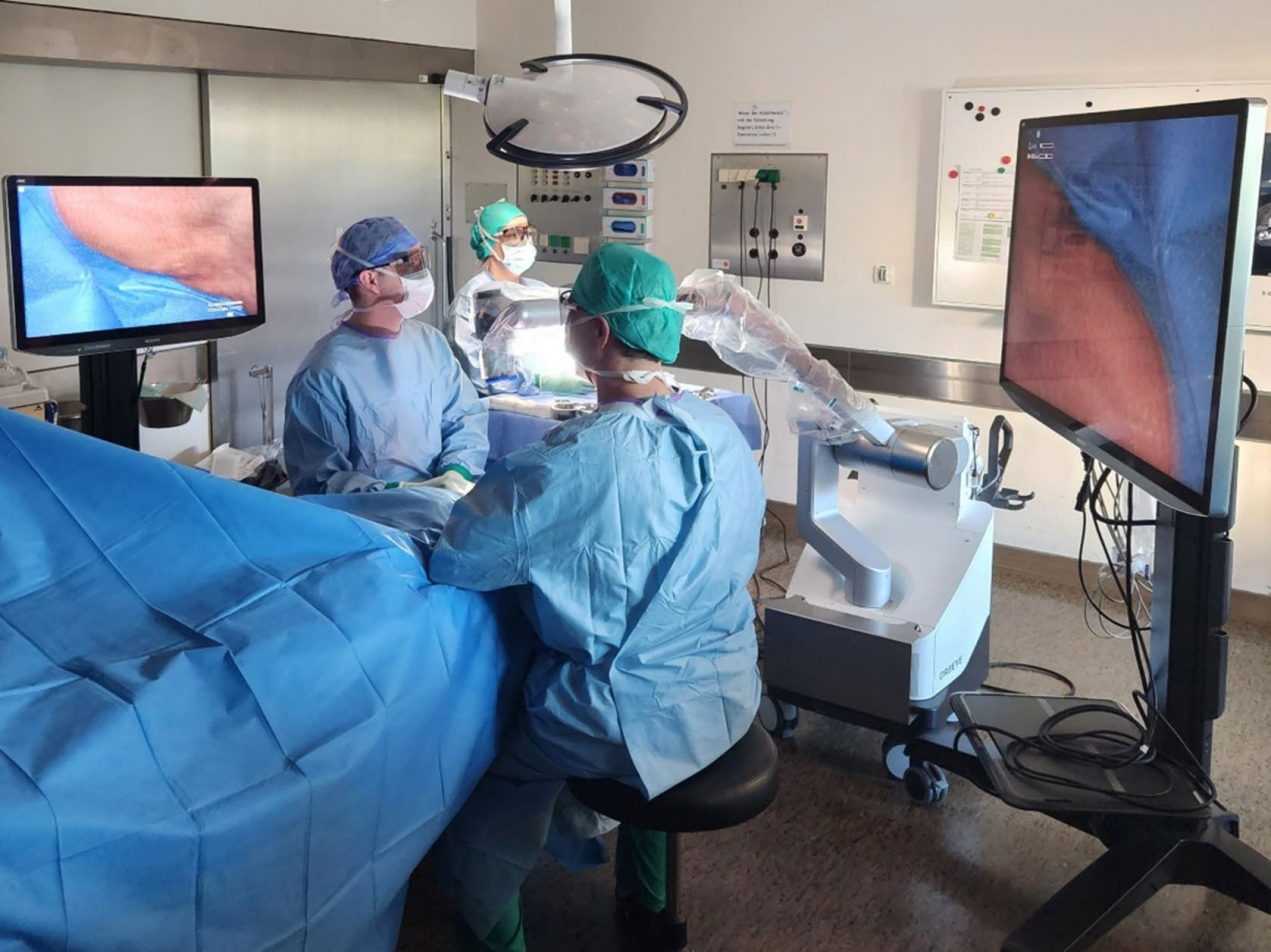
Favored positioning of the exoscope in head and neck surgery. This image, based on the schematic drawing in Fig. **2**a, shows the simulated arrangement in the operating room, which facilitates the shortest distance between the surgeons and the monitors. For optimal visualization, the operating room should be darkened. In this scenario, the main surgeon is seated on the patient’s right side with a direct view of the main monitor. The scrub nurse is positioned near the main surgeon on the patient’s right side

Before the arrangement of the camera system and the monitors, it is important to consider the sterile field and the required walking paths. During reconstructive head and neck surgery, the camera arm is positioned cranially to the patient’s headrest, ensuring optimal ergonomics for the operating team (Fig. 2a, b). The sterile camera is adjusted above the surgical field to provide precise visualization for microvascular anastomoses. For instance, Figs. 2a and 3 show the favored exoscope setup for an anastomosis performed on the patient’s right-hand side, which allows the shortest distance to the monitors for optimized visualization. The set-up of the monitors and the camera system and the position of the main surgeon and scrub nurse can be mirrored, if an anastomosis is performed on the contralateral (left) side of the patient.

Dual monitors facilitate visualization for surgeons positioned on either side of the patient’s head. If two monitors of different sizes are used, the larger monitor should be arranged in the viewing field of the main surgeon performing the anastomoses, considering the localizations and side of the anastomoses. Caudal positioning of the exoscope is not feasible in reconstructive head and neck surgery, especially with two team approaches. During the use of the exoscope, the operating room should be darkened, and all team members must wear 3D glasses. At the time of anastomoses, the camera itself is sterilely covered and positioned just above the surgical field by the surgeon. This also allows easy manual adjustments of the camera position, focus, and viewing field.

### Statistical analysis

Data were collected in Microsoft Excel (Microsoft Corporation, Redmond, WA, USA), and statistical analysis was performed using IBM SPSS Statistics, Version 29.0 (IBM Deutschland GmbH, Ehningen, Germany). Continuous variables were displayed as mean or median values including standard deviations (SDs). Categorical variables were calculated as frequencies and percentages. Patients were followed-up until the last visit or date of death. The observation period ended on March 15, 2024. Overall survival (OS) was defined as the date of surgery until the last visit or date of death, whereas disease-free survival (DFS) was calculated from the date of surgery until disease progression, recurrence, last visit or the date of death. Comparisons between study groups were performed using independent t-tests for normally distributed variables. Categorical data were analyzed using chi-squared tests. *P*-values smaller than 0.05 were denoted as significant.

## Results

### Patient characteristics

Of the 111 patients included during final analysis, the median age was 67 years (SD, 12.82; range: 18–92 years), with 56 males (50.5%) and 55 females (49.5%). Patients underwent surgery for the resection of oral cavity cancer, cervical lymph node resection, and reconstruction using microvascular free tissue transfer, performed by seven experienced head and neck surgeons. Overall, 54 patients presented with stage I–II disease, according to the eighth edition of the Union for International Cancer Control (UICC), and 57 had locally advanced disease (UICC stage III–IV). The average length of hospital stay was 15.6 days (SD, 9.94; range: 5–86 days), and patients spent an average of 3.3 days (SD, 9.43; range: 0‒81 days) in the intensive care unit (ICU) following surgery. Median OS was 16.53 months (SD, 6.82), and DFS was 15.70 months (SD, 7.11). Details of cohorts 1 and 2 are displayed in Table 1.

**Table 1:** Overview of patient details in cohorts 1 and 2

	Cohort 1: Intraoperative use of exoscope (*N* = 55)	Cohort 2: Intraoperative use of microscope (*N* = 56)
Median age (, years)	69 (SD, 12.43)	65 (SD, 12.96)
Sex		
Male (n, %)	28 (50.9%)	28 (50.0%)
Female (n, %)	27 (49.1%)	28 (50.0%)
Mean duration of hospital stay (days)	13.76 (SD, 5.6)	17.3 (SD, 12.67)
Mean duration of ICU stay (days)	2.18 (SD, 3.11)	4.48 (SD, 12.87)
Mean surgical time (hours)	8:33 (SD, 2:18)	9:09 (SD, 2:27)
Number of cases with surgical time > 10 h (n, %)	13 (23.6%)	18 (32.1%)
Complications grade IIIb within 14 postoperative days (n, %)	8 (14.5%)	9 (16.1%)
Median overall survival (months)	14.19 (SD, 4.72)	21.72 (SD, 7.64)
Median disease-free survival (months)	12.71 (SD, 5.23)	21.22 (SD, 7.71)

*SD*, standard deviation. *ICU*, intermediate care unit

The average surgical time for primary oral cancer procedures comprising microvascular reconstruction and anastomoses was 8 h 51 min (SD, 2:23; range: 4:53–15:15 h). The surgical time exceeded 10 h in 31 (27.9%) of the 111 surgical cases. The mean surgical time was comparable between cohort 1 (mean: 8:33 h; SD, 2:18; range: 04:53–13:56 h) with 13 cases (23.6%) lasting longer than 10 h, and cohort 2 (mean: 9:09 h; SD, 2:27; range: 5:20–15:15 h) with 18 cases (32.1%) exceeding a surgical time of 10 h. No difference in the average surgical time between cohort 1 and 2 was evident (t-test, *p* = 0.181).

Free microvascular transplants included radial free flap (*N* = 66, 59.5%), fibula free flap (*N* = 34, 30.6%), anterior vascular thigh flap (*N* = 8, 7.2%), deep circumflex iliac artery bone flap (*N* = 2, 1.8%), and osteocutaneous scapular flap (*N* = 1, 0.9%). One arterial anastomosis and one venous anastomosis were performed in 76 patients; 35 patients underwent microvascular free flap comprising one arterial anastomosis and two venous anastomoses. Arterial anastomoses were routinely performed by suturing (i.e., Prolene 8-0 (Ethicon, Johnson & Johnson Medical N.V., Belgium)). Of 146 venous anastomoses performed in 111 patients, 116 were anastomosed using Coupler devices and 30 were sutured.

Most patients (*N* = 97) were administered the standardized postoperative anticoagulation protocol using 2,800-3,800 I.E- anti-Xa (depending on body weight) subcutaneously twice daily for seven days, and once daily thereafter until full mobilization. The standard protocol was adjusted in 14 patients who received their pre-existing anticoagulant medication due to a pre-existing health problem, and restarted their long-term anticoagulant medication postoperatively.

### Complications during use of exoscope or conventional microscope

The overall rate of grade IIIb complications within 14 days postoperatively was 15.3% (*N* = 17 of 111 patients). Of those 17 patients, three patients were administered an altered anticoagulation protocol due to an anticoagulant agent in their home medication. Further analyses were conducted to compare the rate of intra- and postoperative complications between the application of the exoscope and the use of the microscope.

In cohort 1 with 55 patients, in whom the 3D 4K exoscope was employed, eight postoperative grade IIIb complications were documented within 14 days of the surgical procedure, which accounted for a postoperative complication rate of 14.5%. Complications occurred at a median of 1.5 postoperative days (SD, 3.50; range: 0–11 days). Of the eight patients with complications, one demonstrated a bleeding from the temporary tracheostoma. The other seven patients had a suspected cervical bleeding or anastomosis insufficiency. Anastomotic insufficiency was evident intraoperatively in four patients, in whom a revision was performed.

In the control cohort (*N* = 56) with intraoperative use of the microscope, nine postoperative complications (16.1%) were documented within two weeks of the surgical procedure. The postoperative complications occurred at a median of 3 postoperative days (SD, 3.5; range: 0–10 days). In six of these nine cases, vascular complications at the sites of the anastomoses were suspected or evident. In two of these six cases, the free transplants were lost and explanted due to an anastomosis failure and the lack of revision potential of a very small vessel diameter of the single transplant vein. While two transplant failures were documented in cohort 2, no transplant failures were recorded in cohort 1. Two postoperative complications occurred due to a cervical bleeding, and one documented grade IIIb complication was a wound healing disorder at the recipient site with an intra-extra-oral fistula without an anastomosis-related complication. This study found no difference in the postoperative complication rate between cohort 1 and 2 (chi-squared test, *p* = 0.832).

### Assessment of the learning curve with the use of exoscope

Further analyses of cohort 1 were conducted to compare the results between the time period of three months shortly after the implementation of the 3D 4K exoscope (T1; *N* = 22) and the later time period (T2; *N* = 33). The average operating time was 8 h 42 min (SD, 2:28; range: 5:05–13:56 h) for T1 and 8 h 9 min (SD, 1:59; range: 4:53–12:07 h) for T2 and thus comparable between the two time periods (t-test, *p* = 0.150). The rate of postoperative grade IIIb complications within 14 days following the intraoperative exoscope application decreased over time from 22.7% (*N* = 5 of 22 patients) during the initial three months to 9.1% in the subsequent time period (*N* = 3 of 33 patients). The decrease in the complication rate indicated a learning curve with the exoscope use over time (chi-squared test, *p* = 0.160). The median time period until the first occurrence of a postoperative complication was 2 days (SD, 4.39; range: 0–11 days) for T1 and day 1 (SD, 0.57; range: 1–2 days) for T2.

## Discussion

Few studies have published their first experiences using the 3D 4K camera system, in particular in the field of neurosurgery [4, 6, 7, 9]. The group by Ahmad et al. analyzed the application of the exoscope in various microsurgical cases, including breast, head and neck, extremity, and lymphedema surgeries, comparing it to the conventional microscope. Their study found no significant differences in operating time, ischemia time, or microsurgical complications [2]. However, the surgeons reported improved ergonomics and favorable handling of the exoscope [2]. Similarly, Schupper et al. reported reduced back and neck pain among surgeons using the exoscope, compared to conventional microscope or loupes [12]. No postoperative complications were observed within 30 days in patients undergoing neurosurgical procedures [9]. Despite these positive findings, limitations exist. For instance, the study by Amoo et al. involved a small cohort of 18 patients [9]. Although these results are promising, the limited number of studies and small sample size indicate the need for further research.

The findings from these studies align with the current analysis, which showed no increase in intra- or postoperative complications through transitioning to the exoscope. This work included a total of 111 patients undergoing reconstructive head and neck surgery. A preclinical randomized trial assessing the learning rate for performing a surgical task found noninferior results for the application of the Orbeye™ compared to the microscope use [13]. However, a previous study reported the need to switch to the use of a conventional ocular-based microscope due to challenges such as impaired hand-eye coordination [6]. This underscores the importance of a learning curve, even in the hands of an experienced surgeon, for the successful implementation of this new technology [9]. In this study, no switches to the conventional microscope were required. From our experience, adjusting to the uncoupling of the visual axis from the surgical field depends on the correct positioning of the camera system and the monitors.

This analysis found no change in operating time, independent of whether the conventional microscope or exoscope was employed. Historical reports estimated that a neurosurgeon may spend up to 40% of the operation time adjusting the traditionally used operating microscope [14, 15]. While it may be difficult to quantify the exact time saving with the use of the exoscope, its flexible and simple camera adjustment is a potential time-saving feature. However, it is important to highlight that optimal handling, alignment, and positioning of the system’s components require training. In contrast to the exoscope, the positioning of the conventional microscope has some potential limitations due to the required optical axis between the patient and the surgeons.

The exoscope offers enhanced visualization of the surgical field and procedural steps for all team members through the monitors. This can improve teamwork and streamline processes. Furthermore, surgical training can be improved by providing excellent visualization of the surgical field for training surgeons who can be actively involved in the procedure. However, several limitations in transitioning to the exoscope use in reconstructive head and neck surgery remain. Previous studies reported impaired visualization for the assistant using the exoscope [8]. This limitation can be mitigated by employing a second monitor, which is particularly crucial in head and neck surgeries, where surgeons are positioned on opposite sides of the patient. The precise positioning and alignment of the camera system and monitors can be more demanding, compared to the set-up of the conventional microscope. Therefore, careful training of the entire surgical team and implementation of a standardized intraoperative set-up are essential for a successful transition.

The study addresses the clinical utility of a cutting-edge technology—the 3D 4K exoscope—in reconstructive head and neck surgery, providing valuable insights to a relatively underexplored area and guiding the adoption of new visualization technologies. The findings are grounded in real-world clinical practice in a high-volume Department of Oral and Maxillofacial Surgery, enhancing their practical relevance. The study included 111 surgical cases undergoing microvascular anastomosis, which is a respectable sample size compared to previous studies in this field. The inclusion of a direct comparison between the exoscope and conventional microscopes provides a clear understanding of the relative benefits and limitations of the new technology in the same surgical context. The evaluation of outcomes over two time periods highlights the learning curve associated with exoscope use, offering practical insights into the transition process.

Despite demonstrating the feasibility of the exoscope in a high-stakes surgical setting, the retrospective nature of the study limits the ability to control for potential confounding factors such as surgeon experience and patient characteristics, which might have influenced the outcomes. Although the sample size was reasonable, the number of cases may be insufficient to detect subtle differences in rare outcomes or complications, which might emerge in larger cohorts. Additionally, the study’s evaluation period may not have been sufficient to fully capture the long-term learning curve and the impact of the exoscope on surgical outcomes. The comparison focused on the Orbeye™ and two specific conventional microscopes, which may limit the generalizability of findings to other exoscope or microscope models with different features.

It is also worth noting that a standardized training protocol was not implemented when transitioning to this new technology at our center. Without formalized training, the speed of adoption and proficiency with the exoscope may vary among surgeons. To ensure consistency in skill acquisition, a standardized training protocol should be established.

While the study indicated a reduction in complications over time, the results might underestimate the challenges faced during the early phase of adoption, which could be more pronounced in less experienced teams or lower-volume centers. We recognize that our evaluation relied primarily on general variables such as surgical time and postoperative complications. Future studies could benefit from more specific metrics such as surgeon comfort during use and the impact on team dynamics, to better assess the full impact of the exoscope use. By addressing these limitations in future studies, including prospective, multicenter designs with formalized training and longer follow-up periods, a more comprehensive understanding of the exoscope’s role in reconstructive surgery can be achieved.

## Conclusion

Our results demonstrate the feasibility of implementing a 3D 4K exoscope in reconstructive head and neck surgery. Improved intraoperative comfort and ergonomics for surgeons, as well as enhanced visualization for the surgical team, are notable benefits. However, the transition requires training and careful integration into surgical workflows. The potential for combining the exoscope with robotic systems in the future is promising but remains speculative at this stage. Further studies with larger patient cohorts and diverse surgical settings are necessary to validate these findings and explore the broader applicability of this technology.

## Data Availability

The data that support the findings of this study are not openly available due to reasons of sensitivity and are available from the corresponding author upon reasonable request.
